# The role of magnetic resonance imaging in the postoperative management of cholesteatomas

**DOI:** 10.1016/S1808-8694(15)31378-1

**Published:** 2015-10-17

**Authors:** Carlos Toyama, Claudia da Costa Leite, Iulo Sérgio Baraúna Filho, Rubens Vuono de Brito Neto, Ricardo Fereira Bento, Giovanni Guido Cerri, Eloisa Maria Melo Santiago Gebrim

**Affiliations:** 1 Specialist, MD, researcher; 2 Associate Professor at FMUSP, Head of the Magnetic Resonance Sector at Fundação Faculdade de Medicina; 3 Specialist, Fellowship in ear surgery; 4 Post-PhD, Assistant Professor at USP; 5 Full Professor at the Department of Ophthalmology and Otorhinolaryngology at FMUSP; 6 Full Professor at the Department of Radiology at FMUSP; 7 PhD, Head of the Computerized Tomography Department at the Radiology Institute at FMUSP. University Hoispital of the Faculdade de Medicina da Universidade de São Paulo

**Keywords:** cholesteatoma, diffusion, mri

## Abstract

Conventional CT and MRI scans have low specificity when it comes to differentiating granulation tissue from relapsing cholesteatoma.

**Aim:**

this paper aims to analyze the use of DWI and delayed post-contrast T1-weighed imaging in the detection of recurring cholesteatomas.

**Materials and method:**

this is a cross-sectional prospective study that looked at 17 cholesteatoma patients postoperatively. All patients underwent diffusion magnetic resonance imaging at 1.5T, T1, T2, and delayed post-contrast T1 and images were produced from both coronal and axial planes. Two radiologists assessed the images and decided consensually that the presence of hyperintensive signal in DWI on T2, iso/ hypointensive signal on T1, and absence of contrast uptake were indicative of relapsing cholesteatoma. Surgical review findings were compared to DWI scans.

**Results:**

eleven of the twelve cases of recurring cholesteatoma presented hyperintensive signal in the DWI scans. None of the patients with granulation tissue in the surgical wound presented hyperintensive signal in the DWI scans. A patient with an abscess in the internal acoustic meatus also presented a hyperintensive signal in the DWI scans. Sensibility, specificity, positive predictive value and negative predictive value were 91.6%, 60.0%, 84.6%, and 75.0%, respectively.

**Conclusion:**

DWI combined with delayed post-contrast T1 SE sequence proved to be useful in the differential diagnosis of granulation tissue and recurring cholesteatoma.

## INTRODUCTION

Computerized tomography (CT) has been the imaging method of choice to perform the initial studies of mastoidectomy surgical cavities. But the images produced can be quite unspecific when the cavity is filled with signal dampers and soft tissues^1^, ^2^, ^3^, ^4^. Studies conducted with conventional magnetic resonance imaging have shown it is impossible to differentiate between residual and relapsing cholesteatoma and post-surgery alterations before revision surgery^5^, ^6^, ^7^.

Other studies reported improved specificity when using T1-weighed sequences with delayed post-contrast enhancement, as granulation tissue is poorly vascularized and contrast uptake occurs in a delayed fashion^8^, ^9^. Cholesteatomas are not vascularized and cannot be enhanced by contrast.

Recent studies using diffusion echo planar sequences (DWI-EPI) reported increased specificity in differentiating cholesteatomas from granulation tissue^10^, ^11^, ^12^, ^13^, ^14^, ^15^, ^16^.

This study aims to prospectively assess patients with surgical history of cholesteatoma using diffusion magnetic resonance imaging and delayed post-contrast enhancement to compare imaging and surgical findings and papers published in the literature.

## MATERIALS AND METHOD

This is a prospective cross-sectional study that included 17 patients (9 males and 8 females) with surgical history of cholesteatoma in the mastoid that had been operated from 4 months to 5 years before the study. MRIs done on the patients between December of 2004 and December of 2006 were analyzed and compared to surgical and pathology findings. The surgical procedures were carried out from two weeks to two months after the MRIs were taken. This study was approved by the Ethics Committee of our institution under permit 807/04

MRIs were obtained using a 1.5 Tesla apparatus with head coil, 200mm field of vision (FOV), and a 256 × 256 matrix. T1 and T2 fast spin echo sequences of 3.0 mm axial and coronal views were taken with the following parameters: 512/11/2 (repetition time/echo time/number of acquisitions), and (4600/130/4). Diffusion echo-planar sequences were also done (TR 9000, b 1000sec/mm) of axial and coronal views with a 220 mm FOV. T1-weighed axial and coronal images with fat saturation were taken 30 minutes after contrast (gadolinium) injection at 0.1mmol per kilogram of bodily weight.

The images were consensually assessed by two radiologists and the results compared against surgical and pathology findings.

## RESULTS

**Figure 2:**
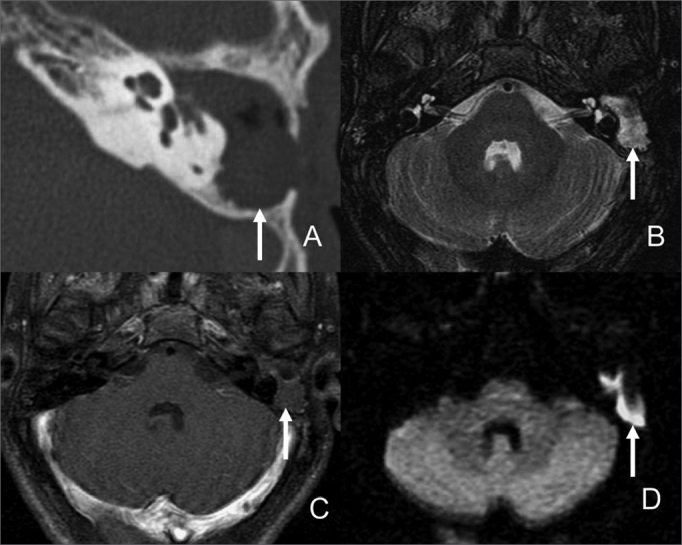
Male patient, 57, with relapsing cholesteatoma in the left mastoid. A- CT scan axial view with unspecific soft tissue in the surgical cavity. B- Axial view on T2; C- Delayed post-contrast axial view on T1; D- Axial view on diffusion imaging. Cholesteatoma resonance images are characterized by hypersignal in diffusion MRI and absence of intravenous contrast uptake.

Surgical findings indicated 12 relapsing cholesteatomas, 11 of which correctly diagnosed through imaging. The lesions presented hyposignal and isosignal on T1, hypersignal on T2 and diffusion imaging in relation to brain parenchyma and did not take contrast up in their inside (Fig. 2). The smallest of the identified lesions measured 5 mm. A 4mm cholesteatoma was missed.

**Figure 1:**
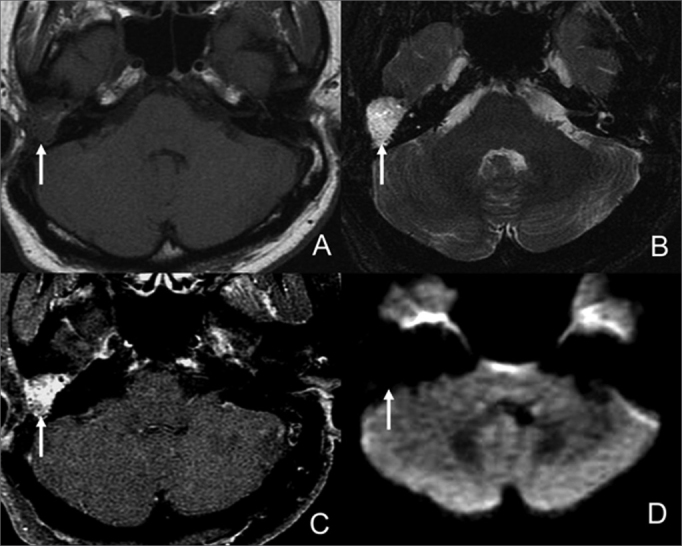
Female patient, 56, operated for cholesteatoma to the right and surgically diagnosed as inflammation in the surgical cavity. A- Axial view on T1 without contrast. B- Axial view on T2; C- Delayed post-contrast axial view on T1; and D- Axial view on diffusion imaging. The surgical cavity is filled by tissue with hypersignal on T2 (B) uptake by intravenous contrast (C) no signal changes on diffusion imaging (D). Contrast uptake and absence of changes in diffusion imaging are indicative of inflammation.

Three patients with granulation tissue in their surgical cavities were diagnosed through MRI and had isosignal on T1, hypersignal on T2, and absence of hypersignal on diffusion imaging and intravenous contrast injection (Fig. 1).

Two nodular lesions with hyposignal on T1, hypersignal on T2, and peripheral uptake of intravenous contrast were observed but were not cholesteatomas. One of them was an abscess located in the inner ear canal produced by a fistula communicating with the surgical cavity. This lesion presented high signal in diffusion imaging and thickened, ring-shaped contrast enhancement.

The other lesion was a mucous cyst in the mastoidectomy cavity that presented linear hypersignal in diffusion imaging and projected itself only in the axial view. Biochemical analysis of the fluid was not performed.

## DISCUSSION

Computerized tomography is currently the imaging technology that provides the best anatomic resolution of temporal bone surgical cavities, but specificity can be quite low when the cavity is entirely or partially filled with signal-attenuating materials and soft tissue, as it may correspond to secretion, granulation tissue, cholesterol granuloma, or relapsing cholesteatoma^1^, ^2^, ^3^, ^4^.

Conventional MRI may differentiate inflamed mucosa and cholesterol granuloma in non-operated patients^17^, but was proven ineffective when differentiating relapsing cholesteatomas^5^, ^6^, ^7^. Reoperation findings in these studies did not present a good radiological-surgical correlation, with values ranging between 50–70%. Sensitivity ranged between 57–79%, specificity between 63–71%, and positive predictive value between 50–78%.

Studies by Williams et al.^8^ and Ayache et al.^9^ reported improved specificity and sensitivity in this differential diagnosis by using delayed (30 minutes) T1-weighed imaging after intravenous contrast injection, as granulation tissue is poorly vascularized and contrast uptake takes longer to occur. Cholesteatomas are not vascularized and do not take contrast up. These findings explain the low specificity found in previous studies in which images were acquired immediately after contrast IV injection. Sensitivity, specificity, positive and negative predictive values were 85.2%, 92.6%, 92.6%, and 85.9% respectively in the paper by Williams and 90%, 100%, 100%, 100%, and 92% in Ayache’s. Using the same principle, a recent study using delayed post-contrast CT also showed improvements in the diagnosis of relapsing cholesteatomas^18^. Sensitivity, specificity, positive and negative predictive values were 75.0%, 60.1%, 88.1%, and 81.8% respectively.

Other studies using diffusion MRI reported high specificity levels in diagnosing cholesteatomas larger than 0.5cm (Table 1)^11^, ^12^, ^15^, ^16^. Vercruysse^16^ reported lower sensitivity levels due to increased prevalence of residual cholesteatomas smaller than 0.5cm in size. Congenital, acquired, and relapsing cholesteatomas present high signals in diffusion imaging and on T2, possibly due to the T2 effect described by Vercruysse^16^, differentiating from post-surgery granulation tissue.

**Table 1:** 

Sens	Spec	PPV	NPV	False -	False +	N	Reference
77%	100%	100%	75%	3 (<5mm)		22	Aikele P. et al AJR 2003; 181:262-26511
86%	100%	100%	92%	1 (2mm)		18	Stasolla A. et al Otol Neurotol 2004;25:879-8412
100%	91%	93%	100%		1 Pó ósseo	24	Dubrulle F. et al Radiology 2006;238: 604-1015
12,5%	100%	100%	72%	6 (<4mm)		45	Vercruysse JP. Et al Eur Radiol 2006; 16:1461-716

Sens: sensitivity

Spec: specificity

PPV: positive predictive value

NPV: negative predictive value

False +: false positive

False -: false negative

N: number of patients

Our study had other findings in agreement with the literature. Smaller cholesteatomas are more easily missed because of the low spatial resolution of diffusion imaging, as seen in one of our cases. Two nodular lesions with high signals in diffusion imaging were also observed but were not cholesteatomas. One inner ear canal abscess connected to a fistula in the surgical cavity had hypersignal in diffusion imaging and, as seen on our case, similar signal characteristics on T1 and T2, except for the intense, thickened aspect it acquired after contrast uptake. Another case of hypersignal on diffusion imaging was an artifact that projected itself as a cyst into the mastoidectomy cavity in the axial view. Diffusion imaging produces artifacts between air and bone in the skull base, usually with a linear shape^19^. This image was not seen in the coronal view, thus supporting the idea that it was indeed an artifact. These two false positive cases of are in agreement with the findings in Dubrulle et al.^15^ in which a diffusion imaging hypersignal false positive was produced from bone powder used to fill up a labyrinthine fistula.

Our study and other papers in the literature indicate that magnetic resonance imaging may be useful in the differentiation of granulation tissue and relapsing cholesteatoma. Nodular lesions with hypersignal in diffusion imaging may be cholesteatomas (Fig. 2), but clinical and surgical data have to be correlated to ensure it is not surgical material or abscess. Diffusion echo-planar imaging still does not produce good sensitivity in the diagnosis of cholesteatomas smaller than 0.5 cm, but technical progress mitigated the presence of artifacts in diffusion imaging with the introduction of fast spin echo^15^ and Propeller^20^ sequences that will possibly improve the detection of primary and relapsing small cholesteatomas in future studies.

## CONCLUSION

Delayed post-contrast diffusion weighed magnetic resonance enables the differentiation between granulation tissue and relapsing cholesteatomas larger than 0.5 cm. Abscess and cholesteatoma cannot be differentiated by diffusion imaging or T1 and T2 signal characteristics, except for the intense and thickened peripheral ring-shaped contrast enhancement.
